# Expression of cell wall related genes in basal and ear internodes of silking *brown-midrib-3*, caffeic acid *O*-methyltransferase (COMT) down-regulated, and normal maize plants

**DOI:** 10.1186/1471-2229-8-71

**Published:** 2008-06-26

**Authors:** Sabine Guillaumie, Deborah Goffner, Odile Barbier, Jean-Pierre Martinant, Magalie Pichon, Yves Barrière

**Affiliations:** 1 INRA, Unité de Génétique et d'Amélioration des Plantes Fourragères, BP6, F-86600 Lusignan, France; 2 UPS CNRS UMR 5546, Chemin de Borde Rouge, F-31326 Castanet-Tolosan, France; 3 Biogemma, ZI du Brézet, 8, rue des frères Lumière, F-63028 Clermont-Ferrand, France

## Abstract

**Background:**

Silage maize is a major forage and energy resource for cattle feeding, and several studies have shown that lignin content and structure are the determining factors in forage maize feeding value. In maize, four natural *brown-midrib *mutants have modified lignin content, lignin structure and cell wall digestibility. The greatest lignin reduction and the highest cell wall digestibility were observed in the *brown-midrib-3 *(*bm3*) mutant, which is disrupted in the caffeic acid *O*-methyltransferase (COMT) gene.

**Results:**

Expression of cell wall related genes was investigated in basal and ear internodes of normal, COMT antisens (AS225), and *bm3 *maize plants of the INRA F2 line. A cell wall macro-array was developed with 651 gene specific tags of genes specifically involved in cell wall biogenesis. When comparing basal (older lignifying) and ear (younger lignifying) internodes of the normal line, all genes known to be involved in constitutive monolignol biosynthesis had a higher expression in younger ear internodes. The expression of the COMT gene was heavily reduced, especially in the younger lignifying tissues of the ear internode. Despite the fact that AS225 transgene expression was driven only in sclerenchyma tissues, COMT expression was also heavily reduced in AS225 ear and basal internodes. COMT disruption or down-regulation led to differential expressions of a few lignin pathway genes, which were all over-expressed, except for a phenylalanine ammonia-lyase gene. More unexpectedly, several transcription factor genes, cell signaling genes, transport and detoxification genes, genes involved in cell wall carbohydrate metabolism and genes encoding cell wall proteins, were differentially expressed, and mostly over-expressed, in COMT-deficient plants.

**Conclusion:**

Differential gene expressions in COMT-deficient plants highlighted a probable disturbance in cell wall assembly. In addition, the gene expressions suggested modified chronology of the different events leading to cell expansion and lignification with consequences far beyond the phenylpropanoid metabolism. The reduced availability of monolignols and S units in *bm3 *or AS225 plants led to plants also differing in cell wall carbohydrate, and probably protein, composition. Thus, the deficiency in a key-enzyme of the lignin pathway had correlative effects on the whole cell wall metabolism. Furthermore, the observed differential expression between *bm3 *and normal plants indicated the possible involvement in the maize lignin pathway of genes which up until now have not been considered to play this role.

## Background

Since shortly after its introduction into Europe, maize has been recognized to be an excellent forage plant and is now the most important annual forage crop in Northern Europe. Despite the fact that maize silage provides roughage with high energy content, large genetic variations in cell wall digestibility have been established between maize genotypes [1]. The lignified grass cell wall is a composite material with cellulose microfibrils, an amorphous matrix consisting of hemicelluloses (mainly glucurono-arabinoxylans) with very little pectins, and phenolics. Phenolics are comprised of lignins, which are essential for the structural integrity of tissues and impart hydrophobicity to vascular elements. They also are comprised of cell wall-linked *p*-coumaric (pCA) ferulic, and diferulic acid derivatives. Lignins are the only cell wall component resistant to micro-organism degradation. Hence, lignin content is a primary factor involved in cell wall digestibility variation. In addition, lignin variable structure and the intensity of cross-linkages between cell wall components also have variable depressive effects on cell wall carbohydrate degradation by microorganisms in the cow rumen. [2-4]. The lignin pathway begins after the shikimate pathway with the deamination of L-phenylalanine into cinnamic acid. Successive steps including hydroxylation and methylation on the aromatic ring lead to production of three monolignols (*p*-hydroxyphenyl, coniferyl, and syringyl alcohols). The latter are polymerized into lignins, giving rise to the three *p*-hydroxyphenyl (H), guaiacyl (G) and syringyl (S) monomeric units. In the maize lignin pathway, caffeic acid *O*-methyltransferase (COMT) is specifically involved in methylation of 5-hydroxy-coniferaldehyde into sinapaldehyde.

Besides the reddish brown pigmentation of the leaf midrib and stalk pith associated with lignified tissues, *brown-midrib *(*bm*) mutants of maize were early distinguished by their lower lignin content and modified lignin structure [5-7]. Among the four non-allelic *bm *mutants, *bm3 *exhibits the strongest phenotype and was early considered to be a powerful model in cell wall digestibility improvement and lignification studies [8-12]. Compared with normal hybrids, cell wall digestibility of isogenic *bm3 *genotypes was increased by about 9 percentage points [1]. Correlatively, and based on several experiments with lactating cows [13-15], the intake of *bm3 *silage by dairy cows was always higher than the intake of normal silage (1.5 to 2.5 kg DM per day). A higher milk yield of cows fed *bm3 *hybrids was also reported in most experiments. Every time this trait was recorded, an increase in body weight was also observed in cattle fed with *bm3 *silage. However, the *bm3 *mutation has adverse effects on agronomic traits such as lower biomass yield and higher susceptibility to lodging and diseases, especially in early maize genetic backgrounds. As a consequence, only a few late maize hybrids are available for farmers on the US market [16-18].

Since the precursory studies done from 1960 to 1970, extensive research has been devoted to maize *bm3 *plants in order to establish the key determining factors in their higher feeding value, with an in- depth description of their specific lignification patterns [2,19-21]. The lignin content of maize *bm3 *mutant plants is reduced by about 25 to 40 %, with a partly correlative reduced content of pCA esters by about 50 %. In the sixties, Kuc et al. [7] established that the frequency of S units was reduced in *bm3 *mutants, and they suspected the occurrence of additional not yet detected units. The thioacidolysis S/G ratio was shown to be reduced from 1.2 – 1.5 in normal plants to 0.2 – 0.5 in *bm3 *plants, and the additional units in lignins were identified as 5-hydroxyguaiacyl (5-OH-G) units [22]. The reduction in pCA esters thus appeared consistent with the preferential acylation of S units by *p*-coumaric acid [23-25]. The content of alkali-releasable FA in *bm3 *mature plants was not altered [2,26]. In younger plants, Marita et al. [27] obtained a greater amount of FA esterified to arabinoxylans in the *bm3 *cell walls. However, similar cross-linking between arabinoxylans (total FA dimers) was obtained.

A nearly null COMT activity has been shown in maize *bm3 *plants [28-30]. Vignols et al. [29] and Morrow et al. [30] later established that *bm3 *mutants had an altered exon 2 of the COMT gene, with different events likely corresponding to different insertions/excisions of a transposable element. Down-regulation of COMT in maize gave plants with similar lignin patterns as observed in *bm3 *mutants [31,32]. In *Arabidopsis*, a lignin devoid of S units was observed due to a mutation in the gene encoding ferulate 5-hydroxylase (F5H) [33,34], while the *Arabidopsis *"*bm3*" mutant lacking the following COMT step in the pathway only had a reduced S content [35]. Only one COMT sequence is described in databases for maize, and a unique COMT in maize would seem to be the most probable hypothesis to date. The presence, even clearly reduced, of S units in *bm3 *lignins then raises a question about a substitutive pathway, and/or a substitutive OMT. In contrast to S unit content, normal ferulic acid content was observed in *bm3 *or AS225 plants, showing that COMT is very likely not involved in its biosynthesis. Ferulic acid biosynthesis in maize is still not clearly understood and different putative pathways have been discussed recently [36]. Based on the data of Nair et al. [37] with the *ref1 *mutant of *Arabidopsis*, ferulic acid could be produced in maize beyond the monolignol pathway. Ferulic acid could be derived from oxidation of the corresponding aldehyde, rather than acting as a precursor of this aldehyde.

In contrast to phenolic compounds, only a few changes in cell wall carbohydrate characteristics were observed in *bm3 *plants. The cellulose content of *bm3 *maize was nearly the same as in normal plants, and the hemicellulose content was reported to be slightly or significantly higher in *bm3 *plants [20]. The composition of hemicelluloses also appeared to be little modified in *bm3 *plants. In plant stalks with just emerging tassels, Marita et al. [27,38] found a higher xylan content (as estimated by xylose) in *bm3 *mutants. Similarly, the xylan substitution with arabinose was a little lower in *bm3 *maize than in normal maize. A minor but significant decrease was also observed for rhamnose in *bm3 *plants [27].

Little data is available on histological comparisons of normal and *bm3 *tissues. Based on scanning electron microscopy observations [39], the sclerenchyma of *bm3 *stem tissues appeared less dense, less rigid and less thick, with larger cell lumens than in normal plants. Moreover, the parenchyma of *bm3 *plants was more rapidly degraded in rumen fluid and the sclerenchyma walls were highly degraded and considerably thinned in *bm3 *plants. In contrast, the sclerenchyma of normal plants was little changed even after 72 hours of incubation in the rumen fluid.

In a study devoted to a comparison of 144 cell wall gene expressions in 20-day-old normal and *bm *plants [40], only seven genes were differentially expressed in the *bm3 *mutant. The seven genes were all over-expressed except for the disrupted COMT. Gene expression was also investigated in young stems of three normal and *bm3 *lines. The latter were harvested 5 and 7 weeks after germination, with a micro-array containing nearly 12,000 ESTs [41,42]. Out of 865 candidate ESTs putatively involved in cell wall digestibility variation, only 53 were differentially expressed between *bm3 *and normal isogenic plants.

The first objective of the present study was to investigate variations in expression of cell wall related genes in older and more lignified tissues of *bm3*, COMT down-regulated, and normal silking maize plants. Investigations were based on ear (young) and basal (old) internodes which differ in their physiological maturity. A second objective was to relate variation in gene expression to the previously described modifications in cell wall components, lignin content and structure of *bm3 *and COMT down-regulated plants. A third objective was to find new factors involved in maize cell wall lignification and assembly, in order to further improve forage maize feeding value. Expression studies were based on a macro-array specifically devoted to cell wall and lignification topics developed in CNRS-UPS Toulouse for such investigations [43].

## Results and Discussion

### Lignin pathway gene expression in normal F2 basal and ear internodes of maize

Older and younger lignification stages were compared in basal and ear internodes of the F2 normal line. All genes known to be involved in constitutive monolignol biosynthesis had a higher expression in younger ear internodes, indicating their higher lignification activity, than in older basal internodes (data not shown). An expression at least three times higher was thus observed for genes encoding phenylalanine ammonia-lyases (PAL), cinnamate 4-hydroxylases (C4H), 4-coumarate:CoA ligases (4CL), caffeoyl-CoA *O*-methyltransferases (CCoAOMT), F5H, COMT, cinnamoyl-CoA reductases (CCR), cinnamyl and sinapyl alcohol dehydrogenases (CAD and SAD). Expressions of hydroxycinnamoyl transferases (HCT) and *p*-coumaroyl-shikimate/quinate 3'-hydroxylases (C3'H) genes were low, raising the possibility of other still unknown homolog genes involved in the pathway. Among the five maize CCoAOMT genes shown to be expressed in maize by Guillaumie et al. [43], the most expressed genes were CCoAOMT5 and CCoAOMT3 in ear and basal internodes, respectively. Previously described CCoAOMT2 [44] was the second most expressed gene in younger internodes and in older ones. Laccase genes were more expressed than peroxidase genes, and more expressed in older internodes, probably confirming the involvement of laccases in monolignol polymerization. However, three peroxidases, including ZmPox2 and ZmPox3, had a reverse expression pattern and were more expressed in younger tissues. These peroxidases could be assumed to be more specifically involved in the first steps of cell wall assembly.

### Lignin content and cell wall digestibility in COMT under-expressed and disrupted plants

Cell wall content was a little lower in AS225 basal internodes, which is likely related to a higher soluble carbohydrate content in this line. Lignin content, estimated as Klason lignin [45] in the cell wall part was reduced by 45 % in both F2*bm3 *and AS225 basal internodes, with a correlative average increase by 30 % in cell wall digestibility, estimated as *in vitro *neutral detergent fiber (NDF) digestibility [46].

### Histochemical staining and lignin pattern alterations in bm3 or AS225 internodes

**Figure 1:**
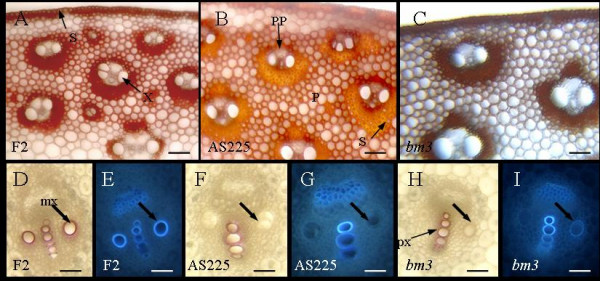
**Histochemical characterization of normal F2, AS225 and F2*bm3 *maize lines**. F2 = normal F2 line; AS225 = COMT-AS line; *bm3 *= F2*bm3 *line. Light microscopy observations of transverse sections in bottom part of ear internode in silking +30 day-old plants stained with Maüle reagent (A-B). Light microscopy observations of transverse sections in bottom part of ear internode in silking plants stained with Wiesner reagent (D, F, H). UV illumination of ear internode bottom part in silking plants (E, G, I). Differences in coloration of lignified tissues between F2, AS225 and F2*bm3 *lines in the presence of Maüle reagent (A-C) are visible. Metaxylem vessels of ear internode transverse sections (D-I) of AS225 and F2*bm3 *plants also had a modified lignification (arrow). x = xylem; s = sclerenchyma; p = parenchyma; pp = protophloem; px = protoxylem; mx = metaxylem. Magnification bar was 100 μm (A-C) or 50 μm (D-I).

At silking + 30 days stage of plant growth, histological observations showed that xylem, sclerenchyma and parenchyma between vascular bundles were lignified. Internodal transverse sections stained with Maüle reagent, which enables to distinguish between S and G units, showed important differences between F2, AS225 and *bm3 *lines (Figure 1A–C). All lignified tissues in F2 plants exhibited a red coloration, showing the presence of S units, whereas in *bm3 *line both sclerenchyma and parenchyma between bundles had a brown coloration, underlying an absence of S units in both of these cell types. In AS225 plants, lignified parenchyma cells stained red, similar to F2 line, but sclerenchyma cells surrounding vascular bundles displayed an orangey color. This difference probably indicated a sclerenchyma-specific reduction of S units in AS225 line and highlighted the fact that using Adh1 promoter directs COMT down-regulation specifically in sclerenchyma tissues [31].

At silking stage, Wiesner staining (Figure 1D, F and 1H) and UV-light (Figure 1E, G, I) observations of the bottom part of ear internode cross sections showed that protoxylem is lignified in all observed lines. Metaxylem vessels are lignified in F2 line but were only weakly lignified in *bm3 *plants and not lignified in AS225 plants.

### Lignin and phenylpropanoid pathway gene differential expression in bm3 or AS225 internodes

**Figure 2:**
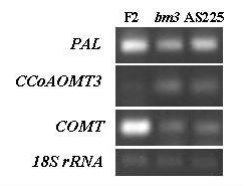
**RT-PCR expression analysis of three genes of phenylpropanoid and lignin pathway**. Transcripts of PAL (2161072.2.1, L77912), CCoAOMT3 (2591258.2.1, AY104670) and COMT (2192909.2.3, M73235) were detected by semi-quantitative RT-PCR in ear internodes from normal, *bm3 *and AS225 silking stage plants. 18S rRNA was used as a quantitative control.

The expression of the COMT gene was, as expected, heavily reduced in COMT-disrupted and down-regulated plants. In younger lignifying tissues of the ear internode, COMT expression was reduced to a nearly null value in *bm3 *plants. Despite transgene, was driven only in sclerenchyma tissues of AS225 plants, COMT under-expression was reduced by 80 % in AS225 ear internodes (Table 1, Figure 2). COMT expression was reduced by 20–25 % in both *bm3 *and AS225 basal internodes (Table 1). More genes were differentially expressed in younger ear internodes than in older basal internodes. In addition, the pattern of gene deregulation was almost similar in *bm3 *and AS225 plants. However, a lower number of genes were differentially expressed in AS225 plants and their expressions were less modified than in *bm3 *plants. Disruption or down-regulation of COMT led to a differential expression of a limited number of genes related to the phenylpropanoid pathway, which were all over-expressed, except a PAL gene in *bm3 *plants. The small number of differentially expressed genes has also been observed in younger growing *bm3 *plantlets [40], illustrating a possible weak apparent co-regulation of lignin pathway genes at these developmental stages. Contrary results were obtained by Shi et al. [41] in a F2/F2*bm3 *comparison, with differentially expressed lignin pathway genes most often under-expressed in *bm3 *plants, except for two cytochrome P450 98A1 (C3'H) over-expressed genes. Observed differences could be related to variable stages of sampling and cropping conditions, and to a different set of investigated genes.

**Table 1:** Phenylpropanoid and lignin pathway genes differentially expressed in maize F2, F2*bm3 *and AS225 basal and ear internodes at silking stage.

	Contig	mRNA	Basal internode			Ear internode		
**Maize phenylpropanoid and lignin pathway related genes**			F2	F2*bm3*/F2	AS225/F2	F2	F2*bm3*/F2	AS225/F2

Phenylalanine/Tyrosine ammonia-lyase (PAL/TAL, MZPAL)	2161072.2.1	L77912	38154	0.50	*0.92*	187353	0.22	*0.66*
Cinnamate 4-hydroxylase 2 (C4H2)	-	CF647652	30820	*1.71*	*1.28*	19988	3.20	*1.80*
4-Coumarate:CoA ligase 2 (4CL2)	-	-	22694	*1.59*	*1.39*	21414	2.36	*1.22*
Caffeic acid O-methyltransferase (COMT)	2192909.2.3	M73235	39113	0.25	0.22	142203	0.05	0.12
Ferulate 5-hydroxylase (F5H)	-	DR966008	13754	3.13	2.34	45662	*0.99*	*0.54*
Caffeoyl CoA O-methyltransferase (CCoAOMT3)	2591258.2.1	AY104670	32846	*1.87*	*1.97*	23740	3.18	2.45
Caffeoyl-CoA O-methyltransferase (CCoAOMT4)	2943966.2.1	AI855419	14245	*1.98*	*1.84*	13947	2.38	*1.54*
Peroxidase (ZmPox2)	2619325.2.1	AJ401275	20634	2.60	*1.58*	10903	4.02	2.66
Peroxidase (4^th ^zinnia DV017512 ortholog)	2763121.2.1	AY110228	15081	*1.75*	*1.27*	10476	2.63	*1.73*
Laccase (Poplar lac3 1^st ^ortholog)	8616263.2.1	BG842157	83977	2.05	*1.79*	46998	3.80	2.79
Laccase (Poplar lac3 3^rd ^ortholog)	2440419.2.1	BT019237	21387	2.08	*1.49*	13702	2.90	*1.85*
Aldehyde dehydrogenase (ALDH, ALDH22A1 ortholog)	2750698.2.1	AY109842	21185	2.05	*1.38*	13749	2.75	*1.75*
Chorismate mutase	2521459.2.4	AY103806	34104	*1.78*	*1.91*	19436	3.85	2.45
Chorismate mutase/Prephenate dehydratase	2950290.2.1	AY109614	19357	*2.09*	*1.54*	18751	2.35	*1.42*
Chalcone flavonone isomerase (ZmCHI1)	3390529.2.1	Z22760	30010	2.79	*1.85*	19524	3.73	2.31

Normalized expression values are given for the F2 line and values for each mutant are expressed as ratios of signal intensity compared to normal plants. Genes were considered to be significantly differentially expressed when expression ratio values were lower than 0.5 or higher than 2.0. Values between the two thresholds are indicated in italics.

Besides the under-expressed COMT gene, the "new" CCoAOMT3 and CCoAOMT4 genes were over-expressed in young ear internodes, while the two currently known CCoAOMT1 and CCoAOMT2 were not differentially expressed (Table 1, Figure 2). CCoAOMT enzymes have a strict affinity for CoA-ester and have no or very little affinity on corresponding acids [36,47-49]. Consequently, it could not be considered that the two CCoAOMT substitute the missing COMT activity in the biosynthesis, of about a 40 % residual S content in *bm3 *plants [2]. The missing COMT activity was probably substituted by other OMT, whether they are over-expressed or not. Plausible candidates include ZRP4-like OMT. Several ZRP4-like OMT genes were indeed shown to be significantly expressed in stalk lignifying tissue [43], and their role is likely not limited to methylation of suberin sub-unit precursors as initially described by Held et al. [50]. Moreover, two ZRP4-like OMT genes were over-expressed in *bm3 *plantlets [40], including the AY108765 ZRP4-like OMT which also have a tendency to be over-expressed in ear internodes of *bm3 *silking plants with a ratio equal to 1.71. Unlike the under-expressed COMT, one F5H gene, whose encoded protein supplies COMT in its 5-hydroxy-coniferaldehyde substrate, has an increased expression in older internodes of *bm3 *and AS225 plants. However, this is not the case in younger ear internodes.

A PAL/TAL gene, which encodes the entry enzyme of the phenylpropanoid pathway, had a greatly reduced expression in F2*bm3 *ear internodes and, to a lesser extent, in basal internodes. However, the expression was not reduced in AS225 internodes (Table 1, Figure 2). A low PAL expression was in agreement with the much lower lignin content of *bm3 *stalk tissues. In contrast to PAL, a C4H and a 4CL gene, whose encoded proteins catalyzed the following steps of the pathway, were over-expressed in *bm3 *young internodes. Also in contrast to PAL under-expression, a chorismate mutase was over-expressed in the youngest internodes of both *bm3 *and AS225 plants, upstream in the phenylpropanoid pathway. In the shikimic acid pathway, chorismate mutase is the enzyme which catalyzes the first committed step in phenylalanine and tyrosine biosynthesis [51,52]. This allows the intra-molecular rearrangement of the enolpyruvyl chain of chorismate to produce prephenate. The chorismate mutase/prephenate dehydratase gene over-expressed in the ear internode of *bm3 *possibly encoded a bifunctional chorismate mutase/prephenate dehydratase as observed by Fischer et al. [53], but its involvement as a precursor supplier for lignin biosynthesis is still unknown.

The ZmPox2 peroxidase gene, whose encoded protein is involved in constitutive lignification [54], had an increased expression in *bm3 *and AS225 plants, especially in younger internodes. Simultaneously with the higher ZmPox2 expression, two laccase genes were similarly over-expressed in *bm3 *young internodes, and one in AS225 plants. These maize laccases were close orthologs to the poplar lac3 gene, whose down-regulation in poplar induced an important alteration of xylem fiber cell walls, with an increase in soluble phenolic compounds, especially in xylem parenchyma cells [55].

According to Nair et al. [37], sinapic and probably ferulic acids in *Arabidopsis *derived from oxidation of the corresponding aldehydes, rather than acting as precursors of those aldehydes. A putative involvement of aldehyde dehydrogenase (ALDH) enzymes in maize ferulate biosynthesis has not yet been established. However, several ALDH genes are expressed in lignifying tissue [43] including the ALDH22A1 *Arabidopsis *ortholog [56,57] over-expressed in COMT deficient tissues, which was also the most expressed ALDH in normal internodes. While the lignin content of *bm3 *maize plants was greatly reduced, their content in ferulates released after alkaline hydrolysis was not modified or slightly increased [2].

The maize chalcone flavonone isomerase (ZmCHI1), described in maize by Grotewold and Peterson [58], was over expressed in *bm3 *and AS225 internodes. CHI catalyzes conversion of chalcone, a yellow pigment synthesized downstream to the coumaroyl-CoA, into naringenin. CHI genes are regulated by MYB transcription factors [58-60], similarly to several other phenylpropanoid-related genes. Because ZmCHI1gene is not involved in the lignin pathway, its over-expression could be interpreted as a possible reaction against over-availability of monolignol precursors.

### Differential expression of transcription or regulation factor genes in bm3 or AS225 internodes

Several transcription or regulation factors, including one ATHB-8 HD-zip III, two Argonaute, and one Shatterproof MAD-box ortholog gene, were over-expressed in both ear and basal internodes of *bm3 *and, to a lesser extent, AS225 silking plants (Table 2). These transcription factors were not differentially expressed in *bm3 *plantlets. However, some of the transcription factors were under-expressed in *bm1, bm2*, and/or *bm4 *plantlets [40]. Homeodomain-leucine zipper (HD-Zip) III proteins are transcription factors which belong to a class of highly related proteins characterized by their expression in (pro)-vascular tissues. ATHB-8 HD-Zip proteins are positively regulated by auxin and promote vascular cell division and differentiation towards the formation of xylem and vascular tissue [61,62]. Argonaute genes are required for post-transcriptional gene silencing and RNA interference, and many of the miRNA which have thus far been identified in plants are predicted to target transcription factors involved in developmental processes [63,64]. An over-expression of an Argonaute gene, as observed in *bm3 *and AS225 deficient COMT plants, is considered to induce a higher repression of target genes. The simultaneous differential expression of Argonaute genes and the ATHB-8 HD-Zip gene could be related. The maize rolled leaf1 gene (rld1) indeed encodes a HD-ZIP III transcription factor, the expression of which is mediated by the miRNA Zmmir166 [65]. SHATTERPROOF (SHP1) MADS-box gene is involved, with SHP2, in lignification of valve margin cells adjacent to the dehiscence zone of *Arabidopsis *siliques [66]. The over-expressed maize SHP1 ortholog gene was the ZmZAG5 gene which has been described with ZmZAG3 [67,68] as an AGL6-like gene (agamous-like). Whether such a SHP maize ortholog could be involved in lignified tissue differentiation or more directly in lignification, is not yet known. However, its deregulation in *bm3 *and AS225 tissue could for the first time illustrate a relationship between this type of regulation factor and cell wall differentiation or lignification in grass plant stalks.

**Table 2:** Cell-wall-related transcription and regulation factor genes differentially expressed in maize F2, F2*bm3 *and AS225 basal and ear internodes at silking stage.

	Contig	mRNA	Basal internode			Ear internode		
**Transcription and regulation factors**			F2	F2*bm3*/F2	AS225/F2	F2	F2*bm3*/F2	AS225/F2

ATHB-8 HD-zipIII	QBN21E06.xg.2.1	CO529337	16199	2.27	*1.87*	14535	2.88	*1.62*
Argonaute	131537.2.170	AY104211	20557	2.67	*1.72*	13525	3.64	2.23
Argonaute	1804958.2.1	AY110984	11026	*1.88*	*1.19*	10027	2.23	*1.30*
SHATTERPROOF (SHP1) MADS-box (ZmZAG5)	L46398.2.1	L46398	20258	2.50	2.07	15008	3.50	2.28

Normalized expression values are given for the F2 line and values for each mutant are expressed as ratios of signal intensity compared to normal plants. Genes were considered to be significantly differentially expressed when expression ratio values were lower than 0.5 or higher than 2.0. Values between the two thresholds are indicated in italics.

### Differential expression of genes related to auxin signaling and tissue patterning in bm3 or AS225 internodes

Several genes related to auxin signaling, and/or probably involved in vessel set up or tissue patterning, were over-expressed in basal or ear internodes of COMT deficient plants (Table 3). This occurrence probably indicated a feedback disorganization of tissues and cell wall assembly when the activity of the key COMT enzyme was missing.

**Table 3:** Cell-wall-related genes involved in auxin signaling and tissue patterning differentially expressed in maize F2, F2*bm3 *and AS225 basal and ear internodes at silking stage.

	Contig	mRNA	Basal internode			Ear internode		
**Auxin signaling and tissue patterning related genes**			F2	F2*bm3*/F2	AS225/F2	F2	F2*bm3*/F2	AS225/F2

Pinoid	2591032.2.1	DR972540	25300	2.23	*1.70*	14555	3.42	2.12
Emb30	2750663.2.1	AY107774	43944	2.76	2.15	91982	*1.48*	*0.77*
Monopteros	2441276.2.1	AY109838	23901	*1.96*	2.03	19267	2.60	*1.85*
Cov1 Integral membrane protein	3562100.2.1	AY112170	29982	*1.94*	*1.51*	18031	2.96	2.08
Cucumisin	7297169.2.1	AY105082	26913	*1.98*	*1.51*	13967	3.47	2.43

Normalized expression values are given for the F2 line and values for each mutant are expressed as ratios of signal intensity compared to normal plants. Genes were considered to be significantly differentially expressed when expression ratio values were lower than 0.5 or higher than 2.0. Values between the two thresholds are indicated in italics.

PINOID (PID) orthologous genes, of which one member was over-expressed in basal and ear *bm3 *internodes, and in ear AS225 internodes, encode serine/threonine protein kinases. PID genes are preferentially expressed in the vascular tissue of developing organs and in xylem parenchyma cells [69], and have been shown to be positive regulators of polar auxin transport [69-71]. The Emb30 gene of *Arabidopsis*, whose maize ortholog is over-expressed in basal internodes of *bm3 *and AS225 plants, is involved in the maintenance of polar auxin transport. In addition, the lack of Emb30 protein induced an irregular and discontinuous venation, with clustered or scattered tracheary elements [72,73]. The Emb30 gene encodes a guanine nucleotide exchange factor (GEF) on adenosine diphosphate (ADP)-ribolysation factor GTPase (ARF-GEF), which is involved in the targeted recycling of PIN1 putative auxin efflux carrier [74]. Its over-expression in *bm3 *and AS225 plants was likely a consequence of tissue assembly with a reduced amount of S units. In addition, one MONOPTEROS (MP) gene ortholog was over-expressed in *bm3 *ear internodes. The MP gene of *Arabidopsis *encodes a transcription factor of the auxin response factor (ARF) family, which is also involved in vascular cell differentiation [75,76]. The *Arabidopsis *recessive mutant "CONTINUOUS VASCULAR RING" (COV1) has a great increase in stem vascular tissue in of inter-fascicular regions [77]. Moreover, it has been established that cov1 affects vascular patterning by a mechanism which is not auxin-dependant [77]. In addition, cov1 mutants were shown to be defective in synthesis, transport, or perception of an inhibitor. The maize COV1 ortholog over-expression in *bm3 *ear lignifying internodes thus corresponded to a higher inhibition in lignified tissue formation, possibly illustrating a feed-back effect resulting from the low availability of S units.

Cucumisins are serine protease of the subtilisin family [78]. Cucumisins are involved in the general protein turn-over as non-selective enzymes, but they also perform specific roles in the processing of precursor proteins to regulate growth and development by limited proteolysis [79]. The over-expressed maize cucumisin in *bm3 *and AS225 ear internodes was the closest ortholog to a zinnia (*Zinnia elegans *Jacq.) cucumisin expressed during xylogenesis [80]. Similarly, a subtilase (XSP1, At4g00230) has been identified from a xylem library of *Arabidopsis *[81]. The role of cucumisins in phenylpropanoid biosynthesis is still unknown. However, it is plausible that several cucumisins could function during autolysis of tracheary elements. A cucumisin gene deregulation in *bm3 *internodes could be in agreement with the lower stiffness of *bm3 *plant vessels.

### Differential expression of transport and detoxification genes in bm3 or AS225 internodes

Four genes involved in transport and detoxification processes were over-expressed in *bm3 *and/or AS225 internodes (Table 4). ATP-binding cassette (ABC) transporters are involved in different physiological functions such as cell signaling, transport of a broad range of substances across membranes and detoxification processes [82,83]. Given their ability to transport a diverse set of small molecules across membranes, and because several ABC transporters had similar expression profiles to known monolignol genes along the *Arabidopsis *growing stem, ABC transporters were assumed to be involved in the secretion of monolignols [52,82,84]. The maize ABC transporter over-expressed in internodes of maize *bm3 *plants could thus be considered as a monolignol transporter. Based on its under-expression in *bm2 *plantlets, it has been assumed to be preferentially involved in coniferyl alcohol transport [40].

**Table 4:** Cell-wall-related genes involved in transport and detoxification processes differentially expressed in F2, F2*bm3 *and AS225 basal and ear internodes at silking stage.

	Contig	mRNA	Basal internode			Ear internode		
**Transport and detoxification process genes**			F2	F2*bm3*/F2	AS225/F2	F2	F2*bm 3*/F2	AS225/F2

ABC transporter	3024030.2.1	DT653269	18994	2.35	*1.81*	13193	3.38	2.11
Glutathione S-transferase II (ZmGST17. Bronze2 like)	3829517.2.1	AF244682	36756	2.43	*1.74*	16243	4.91	2.50
Glutathione S-transferase III (ZmGST22. Bronze2 like)	2441327.2.1	AF244687	19055	*1.75*	2.32	20836	2.76	2.61
UDP-glucosyltransferase like	3748389.2.1	CG357903	83751	2.23	*1.96*	64132	2.80	*1.91*
MtN21 Nodulin-like protein	3203235.2.1	CO529888	18082	2.21	*1.89*	11373	3.24	2.31

Normalized expression values are given for the F2 line and values for each mutant are expressed as ratios of signal intensity compared to normal plants. Genes were considered to be significantly differentially expressed when expression ratio values were lower than 0.5 or higher than 2.0. Values between the two thresholds are indicated in italics.

Maize bronze2-like genes encode glutathione S-transferases (GST). The latter are carrier proteins [85] which deliver anthocyanins from the biosynthesis site to the tonoplast membrane, where an appropriate ABC-type transporter moves the pigment into the vacuole [86]. Because several ABC transporters have a substrate preference for glutathione conjugates [82,83,87,88], a coupled activity could be assumed between the two over-expressed ZmGST17 and ZmGST22 and the over-expressed ABC transporter. The much reduced COMT activity and S unit formation could induce the accumulation of phenolic compounds that have to be delivered into the vacuole as a detoxification process.

Plant glucosyltransferases belong to a multigene family, which are likely differentially regulated in different biosynthesis pathways and in response to a range of environmental stimuli. Glucosyltransferases have relative substrate specificity because of their extended plasticity towards metabolites of related structure [89]. They are involved in the biosynthesis of secondary plant metabolites and metabolization of xenobiotics. They are known in particular to have activity on both flavonoids and *p*-hydroxycinnamate derivatives. In addition, a grapevine UDP-glucose:flavonoid 3-*O*-glucosyltransferase, has been shown to be homolog to the maize bz1 encoded protein [90]. The role of the protein encoded by the differentially expressed UDP-glucosyltransferase-like gene is still unknown, but it could possibly be involved in detoxification and/or transport processes.

The role of nodulin MtN21-like genes is not yet understood, but the presence of seven transmembrane domains and structural homologies with bacterial multidrug exporters might suggest a role in a transport function. The maize nodulin MtN21-like gene over-expressed in *bm3 *and/or AS225 internodes was found to be orthologous to a zinnia nodulin MtN21-like gene expressed during xylogenesis. Consequently, the maize nodulin MtN21-like gene is probably involved in transport of a component related to vascular tissue assembly.

### Differential expression of cell wall associated protein genes in bm3 or AS225 internodes

Several arabinogalactan proteins (AGP) and AGP-like proteins, which belong to a class of hydroxyproline-rich glycoproteins (HRGP), have been shown to be involved in plant xylogenesis [91-93]. Some of these proteins were thus highly preferentially expressed in differentiating xylem compared with phloem [93,94]. Out of the four genes encoding cell wall proteins which were differentially expressed in *bm3 *or AS225 internodes (Table 5), ZmRCP1 and ZmRCP2 showed especially high over-expression ratios (up to five times higher) in ear internodes. The large differential expression values in COMT deficient plants likely indicate that these AGP genes are relevant targets in understanding maize cell wall assembly.

**Table 5:** Cell-wall associated protein genes differentially expressed in maize F2, F2*bm3 *and AS225 basal and ear internodes at silking stage.

	Contig	mRNA	Basal internode			Ear internode		
**Cell-wall associated proteins**			F2	F2*bm3*/F2	AS225/F2	F2	F2*bm3*/F2	AS225/F2

Arabinogalactan protein (AGP, ZmRCP1)	AB021175.2.1	AB021175	49374	2.26	2.42	20741	5.49	4.01
Arabinogalactan protein (AGP, ZmRCP2)	AB021176.2.1	AB021176	41219	2.34	*1.62*	17816	4.76	3.20
Glycine rich protein (GRP)	1716296.2.7	BT018002	161370	2.35	0.44	223451	2.53	0.42
Proline rich protein (PRP)	131537.2.203	AY105945	4829	*1.40*	*0.80*	28975	0.20	*1.33*

Normalized expression values are given for the F2 line and values for each mutant are expressed as ratios of signal intensity compared to normal plants. Genes were considered to be significantly differentially expressed when expression ratio values were lower than 0.5 or higher than 2.0. Values between the two thresholds are indicated in italics.

One glycine-rich protein (GRP) was the only investigated gene with an over-expression in *bm3 *internodes and an under-expression in AS225 internodes. This significant interaction could be related to the lack of complete isogenicity of F2 and AS225 genetic backgrounds, but also to the fact that COMT down-regulation in AS225 was directed specifically in sclerenchyma tissues. GRP genes encode a group of cell wall structural proteins which are particularly expressed in xylem tissues [95,96]. The differentially expressed GRP gene is the closest ortholog of the rice Osgrp-2 gene (AF010580) which possesses sequence elements conferring vascular-specific expression [97].

The differentially expressed proline rich protein (PRP) was one of the rare genes significantly under-expressed in COMT-deficient ear internodes. PRP are considered to be structural components of the cell wall and are abundant in fibers and xylem [98]. Vignols et al. [99] have thus described the maize ZmPRP, which is expressed in vascular tissue, mainly in cells related to xylem development, but not in phloem cells. However, the differentially expressed PRP and ZmPRP are very different genes, with only 15 % identity. The protein encoded by the under-expressed PRP gene is a basic PRP, with 26 % of proline amino acid and a peptide signal allowing protein secretion in the cell wall. It also presents a six-times repeated PPVTGPP(KG)(P)VTYPP motif, which is different from the classic PRP motif [PPVX(K/T), with X being Y, H, or E]. This PRP, which is probably involved in cell wall formation, appeared to be an original and possibly new type of cell wall PRP.

### Differential expression of genes involved in nucleotide sugar interconversion and cell wall carbohydrate metabolism in bm3 or AS225 internodes

The nucleotide sugar interconversion pathway comprises a set of enzymatic reactions by which plants synthesize activated monosaccharides as precursor elements of cell wall polysaccharides [100]. Nucleotide sugars are thus substrates used for the elongation of carbohydrate chains by UDP-glycosyltransferase [101,102]. Cellulose is synthesized by an enzyme complex associated with the plasma membrane, using UDP-D-glucose as a precursor. While UDP-D-glucose is also a precursor of hemicelluloses, hemicellulose polysaccharides are formed in the Golgi apparatus and are exported to the external surface of the membrane in Golgi vesicles [103].

Seven genes of the nucleotide sugar interconversion pathway, which belonged to five multigene families, were over-expressed in *bm3 *and/or AS225 plants (Table 6). Sucrose synthases catalyze a reversible reaction giving UDP-D-glucose from sucrose and uridine diphosphate (UDP). UDP-D-glucose dehydrogenases convert UDP-D-glucose into UDP-D-glucuronic acid (UDP-D-GlcA). UDP-D-GlcA is afterwards the substrate of UDP-D-GlcA decarboxylases, giving UDP-D-xylose, which is then converted into UDP-L-arabinose in a reversible reaction catalyzed by UDP-D-xylose 4-epimerases [102]. GDP-D-mannose 4,6-dehydratase catalyzes the irreversible conversion of GDP-D-mannose into 4-keto-6-deoxy-GDP-D-mannose, then allowing the synthesis of GDP-fucose [100,102]. In plant stalks with just emerging tassels, a decrease in mannose content has been observed in *bm3 *plants [27], which could be related to over-expression of GDP-D-mannose 4,6-dehydratase.

**Table 6:** Nucleotide sugar and cell-wall carbohydrate metabolism genes differentially expressed in maize F2, F2*bm3 *and AS225 basal and ear internodes at silking stage.

	Contig	mRNA	Basal internode			Ear internode		
**Nucleotide sugar and cell wall carbohydrates**			F2	F2*bm3*/F2	AS225/F2	F2	F2*bm3*/F2	AS225/F2

Sucrose synthase (ZmSPS5a)	3748394.2.2	M97550	66356	*1.08*	*0.86*	36451	2.17	*1.50*
Sucrose synthase (ZmSus1)	131537.2.202	L22296	62970	*1.0*	*1.38*	92642	*0.96*	2.10
UDP-D-glucose dehydrogenase	2750995.2.1	AY103689	35321	2.18	*1.56*	17554	3.83	2.49
UDP-D-glucose dehydrogenase	2405245.2.2	AF457980	34777	*1.95*	*1.85*	27367	2.54	2.48
UDP-D-glucuronic acid decarboxylase (AtUXS2 ortholog)	3042304.2.1	AY106346	14116	5.62	2.53	12568	*1.59*	4.34
UDP-D-xylose 4-epimerase (mur4 ortholog)	3070703.2.1	AY106136	24293	2.33	*1.32*	27434	3.68	*1.33*
GDP-D-mannose 4.6-dehydratase (mur1 ortholog)	2621786.2.1	AF134575	62635	2.94	2.92	181413	*1.32*	*0.59*
α-5-expansin	3696569.2.1	AF332173	21885	2.40	*1.39*	13826	3.36	2.22
β-5 expansin	2419405.2.2	AF332178	18565	2.39	2.17	17248	2.46	*1.85*
Xyloglucan endotransglycosylase (XTH)	8010573.2.1	U15781	25201	2.32	*1.68*	21669	3.29	*1.75*
Endo-1.3–1.4-β-D-glucanase (ZmGnsN1)	2192481.2.2	AF072326	27390	*1.69*	*1.88*	19511	3.03	2.29
Endoglucanase (endo-1.3-β-glucanase)	3713002.2.1	AY109289	44167	2.30	*1.43*	23557	3.66	2.08
Endoglucanase (endo-1.3-β-glucanase)	2493751.2.1	AY111608	36875	2.43	*1.62*	16100	4.90	3.06
Exoglucanase (β-D-glucan exohydrolase II)	2437739.2.2	AF064707	15307	2.39	*1.60*	15315	2.39	*1.35*
Xyloglucan fucosyltransferase (AtFUT1 ortholog)	3670899.2.1	AW287792	23393	*1.84*	*1.77*	16818	3.21	*1.91*
Xyloglucan fucosyltransferase (AtFUT2 ortholog)	5351892.2.1	AY112574	34273	2.02	*1.27*	21944	2.74	*1.83*
Pectate lyase	3198766.2.1	CG140821	35864	2.18	2.19	78008	2.17	2.87
Cellulose synthase (ZmCesA-12)	QBS7b05.xg.2.1	AY372246	45366	*1.88*	*1.41*	42685	2.14	*1.54*
Cellulose synthase (ZmCesA-9)	2441542.2.1	AF200533	14649	*1.95*	*1.33*	15755	2.22	*1.58*

Normalized expression values are given for the F2 line and values for each mutant are expressed as ratios of signal intensity compared to normal plants. Genes were considered to be significantly differentially expressed when expression ratio values were lower than 0.5 or higher than 2.0. Values between the two thresholds are indicated in italics.

Several genes involved in cell wall modification during cell growth and vascular element formation were differentially expressed between normal and COMT deficient plants (Table 6). Expansins are involved in the disruption of hydrogen bounds between cellulose microfibrils and cross-linking glycans in the cell wall. At least five different expansin genes are expressed in differentiating tracheary elements of zinnia. These genes were associated with the intrusive growth of protoxylem and with the differentiation of mesophyll cells into tracheary elements [80]. Xyloglucan endotransglycolase/hydrolases (XTH) and glucanases modify the structure of the cell wall so that it becomes more responsive to primary wall-loosening events [104,105]. Xyloglucan binds non-covalently to cellulose, coating and cross-linking adjacent cellulose microfibrils. The resulting network is considered to be the major tension-bearing structure in the primary wall [106]. Cleavage of xyloglucan chains by hydrolytic enzyme leads to rapid wall loosening, but also induces important risks of structural failure in the absence of concomitant reinforcement. XTH, capable of splitting and reconnecting xyloglucan molecules in a new position, help to satisfy the contradictory needs of growing and/or differentiating tissues [106]. Exo and endoglucanases are involved in specific degradation of cell wall β-glucans in auxin-mediated cell elongation [107,108]. ZmGnsN1, which was over-expressed in ear internodes of *bm3 *and AS225 plants, encodes an endo-1,3–1,4-β-D-glucanase first found in maize seedling growing tissues [107-109]. The two other over-expressed endoglucanases were ortholog to β-1,3-glucanase, which are also considered to be involved in hydrolysis of cell components and could be induced by hormone or wounding signals [110]. An exoglucanase was also overexpressed in *bm3 *internodes which has been shown to preferentially hydrolyze the non-reducing terminal glucosyl residue from 1,3-β-D-glucans [111].

In maize, most hemicelluloses are arabinoxylans, with a few xyloglucans which are predominant in primary walls of dicotyledons. Xyloglucans have a backbone composed of β-1,4-glucose with up to 75% of the residues substituted with mono-, di-, or triglycosyl side chains. α-1,2-Xylosyl residues, linked to O6 of β-1,4-glucose, are partly further substituted by β-1,2-D-galactosyl. The latter can be further substituted by α-1,2-linked fucosyl residues [112]. This last step is catalyzed by xyloglucan fucosyltransferases and two genes encoding members of this multigene family were over-expressed in ear internodes of COMT deficient plants.

Cell autolysis is a key event in tracheid formation. A maize gene ortholog to a pectate lyase (ZePel) expressed in the zinnia model system was found to be over-expressed in ear internodes of *bm3 *and AS225 plants. ZePel was strongly expressed at a very early stage of tracheary element induction, and was related to the presence of auxin. Moreover, in young zinnia stems, ZePel expression was associated with vascular bundles [113]. The over-expression of this gene in *bm *maize is likely an indication that this type of enzyme is involved in monocotyledon plant lignification.

Two cellulose synthase genes were over-expressed in *bm3 *or AS225 internodes whereas no drastic modification was described in cellulose content in *bm3 *plants. Interestingly, ZmCesA-9 and ZmCesA-12 are respectively homolog to PtrCesA5 [114] and HvCesA8 [115] genes, highly expressed in developing xylem and stem undergoing significant secondary cell wall biosynthesis.

## Conclusion

Disruption or down-regulation of COMT gene induced a correlative differential expression of only a few genes involved in the lignin pathway. These genes were all over-expressed, except for an under-expressed PAL gene. As a consequence, deregulation of maize COMT did not clearly highlight major co-regulation of lignin pathway genes. Similarly, no over-expressed phenylpropanoid related gene could explain that as much as 40 % of S units were still present in *bm3 *lignins, with no COMT expression. Syringyl alcohol thus probably resulted from methylation by other OMT which were sufficiently available. One ZRP4-like OMT gene, which was shown to be over-expressed in maize *bm3 *plantlets [40] and which also had a tendency to be over-expressed in the *bm3 *ear internode, could be a candidate for the replacement of the lacking 5-hydroxyconiferaldehyde methyltransferase activity.

Differential gene expressions in *bm3 *and/or AS225 internodes highlighted a probable disturbance in cell wall assembly and/or a possibly modified chronology of the different events leading to cell expansion and lignification with consequences far beyond the phenylpropanoid metabolism. The reduced availability of monolignols and S units led to deeply different walls, with probably greater differences in carbohydrate composition than currently considered. During cell wall assembly, biosynthesis of phenylpropanoid, protein, and carbohydrate constituents appeared to be inter-dependent, and the deficiency in a key-enzyme of the lignin pathway had consequences on the expression of several genes involved in cell wall metabolism. Apart from a PRP gene in *bm3 *plants and a GRP gene in AS225 plants, all non-lignin-pathway differentially expressed genes were also over-expressed. Furthermore, the lower number of deregulated genes in AS225 plants than in *bm3 *plants could result, in addition to the difference in genetic background of the two progenies, from the sclerenchyma-limited expression of the COMT-antisens constructed gene.

The differential expression of genes in COMT-deficient plants is likely an indicator of their involvement in cell wall assembly, and some of these genes are probably new relevant targets for silage and biofuel maize breeding. While the transcription factors involved in regulation of lignin biosynthesis and deposition in maize are not yet clearly understood, the differential expression of one ATHB-8 HD-zip III, two Argonaute, and one Shatterproof MAD-box orthologous genes between *bm3 *and normal plants could be the first evidence of their role in the cell wall metabolism. Similarly, maize COV1 ortholog could be assumed to be involved in lignified fiber tissue patterning. Finally, the comparison of gene expression in silking normal and *bm3 *plants highlighted the possible involvement in the constitutive lignin pathway of not yet considered genes, such as ZRP4-like OMT.

## Methods

### Plant material and RNA extraction

The well known normal F2 line, originating from the Lacaune landrace, and its isogenic F2*bm3 *(7 backcrosses) were used with the COMT-AS (AS225) down regulated progeny previously described by Piquemal et al. [31] and Pichon et al. [116], which has only one backcross with the recurrent parent F2 (75 % of F2 genetic background). The use of the Adh1 promoter directed COMT down-regulation specifically in sclerenchyma tissues, as shown by Maüle staining [31]. Hence, there was a different topology of modified tissues between COMT down-regulated plants and *bm3 *COMT-disrupted mutant plants. Plants were cropped in a greenhouse during the spring of 2004 at Lusignan (France), in pots with a mix of sand and compost, and were fed with usual nutritive solution.

Nine plants were harvested at silking stage, just before pollen shedding. The plants were divided into three pools of three plants each for transcriptome analysis. The first elongated basal internode and the internode below the node bearing the ear (ear internode) were sampled separately from each pool. Nodes and leaf sheaths were eliminated, and internodes were immediately frozen in liquid nitrogen. Total RNA was isolated from each set of frozen internodes (5 g) by a method adapted from Ragueh et al. [117]and developed in Guillaumie et al. [43]. Internodes of grasses elongate in acropetal staggered succession, with an intercalary meristematic zone at the base of each internode which remains active until the final stage of elongation [118,119]. The basal internode is then physiologically older than the ear internode, and its lignification is more advanced than the more newly extended and fully lignifying ear internode [120-122].

### Histochemical Staining of Lignins

Stem sections were cut with a vibratome from silking stage plants and silking + 30 days plants grown in the greenhouse. Wiesner and Maüle reactions were performed according to standard protocols [123]. Sections were observed using an inverted microscope (Leitz DMRIBE, Leica Microsystems, Wetzlar, Germany) with bright-field optics or epifluorescence illumination. Images were registered using a CCD camera (Color Coolview, Photonic Science, Milham, UK) and characterized through image analysis (Image PRO-Plus, Media Cybernetics, Silver Spring, MD). Wiesner reagent specifically reacts with cinnamaldehyde side chains in lignins and underlines the presence of lignins with a pink coloration. Lignified cell walls of xylem appear blue under UV illumination.

### Maize cell wall macro-array construction, membrane hybridization, and data analysis

The maize cell wall macro-array, which consists of gene-specific tags (GSTs) for 651 genes, was described in detail by Guillaumie et al. [43]. Spotted genes were chosen firstly as maize orthologs of genes expressed during secondary cell wall formation of zinnia (*Zinnia elegans *Jacq.) tracheary elements [80] and secondly from a keyword strategy developed from sequences previously described as involved in primary and secondary cell wall biosynthesis and assembly for all species throughout the plant kingdom. Maize orthologous genes were found from BLAST (blastn and tblastx) searches [124] performed against GenoPlante-Info databases [125].

Membrane hybridizations and data analyses were achieved according to a protocol adapted from Pesquet et al. [80] and developed in Guillaumie et al. [43]. Each GST was diluted to a final concentration of 0.5 mg/ml and then denatured in 50% DMSO. Controls were added in separate 384-well plates. One plate included 384 Tris-EDTA, pH 8.0 (blank background control). Another plate had 30 NPT II fragments (a positive hybridization control), 20 pBluescript plasmids (unspecific hybridization control), and 18 ubiquitin fragments (positive control). All fragments were spotted onto a 20 × 20-cm Nytran SuPerCharge nylon membrane (Schleicher and Schuell, Keene, NH, USA) using a BioGrid spotting robot (BioRobotics) in a 4 × 4 grid organization. Each gene was spotted twice in two different membrane grids corresponding to four replicates. cDNA probes were synthesized according to Guillaumie et al. [43] from 10 μg of total RNA for each sample. Membranes were placed in a PhosphorImager cassette (Molecular Dynamics, Amersham- Pharmacia) for 72 h and scanned at 50 mm/pixel by a Storm 820 scanner (Amersham-Pharmacia).

Data analysis was performed according to Pesquet et al. [80] and Guillaumie et al. [43]. Macro-array gridding and gene expression levels were measured with ImageQuant 5.0 software (Molecular Dynamics, Amersham-Pharmacia) using 4 × 4 grids. Three independent hybridizations, each corresponding to one of the three groups of ear or basal internodes, were performed for normal, AS225 and *bm3 *plants. Reproducibility of raw signal intensity values on the membrane was first verified inside each grid and between the two grids for each investigated gene. According to Pesquet et al. [80] and Guillaumie et al. [43], two threshold values were thus defined so that 95% of the ratios between raw values of two replicates would be within the 0.5–2.0 interval. Aberrant values giving ratios outside of this interval were discarded. Normalization was performed based on the blank background, unspecific hybridization and positive controls (Tris-EDTA pH8, pBluescriptII, ubiquitin and kanamycin-NPTII). Macro-array reproducibility was further investigated by comparing normalized spot intensity values from the three independent hybridizations performed on three independent membranes. As was developed for reproducibility within the membrane, the investigations on the threshold values showed that more than 95% of the ratio values between independent hybridizations were confined within a twofold limit. Out of range values were discarded and 96–99% of the ratios between replicates were found between the two 0.5–2.0 threshold values. Expression data were estimated as the average of normalized intensity signal values of replicates, and comparisons between F2*bm3 *or AS225 with the normal F2 line were based on expression value ratios. According to Pesquet et al. [80] and Guillaumie et al. [43], genes with more than a twofold expression ratio in F2*bm3 *or AS225 compared with F2 were considered as differentially expressed genes. In addition, a variance analysis based on elementary normalized signal intensity values, followed by a bilateral Student t test (risk levels of P < 0.05), was performed. All genes that were considered differentially expressed were also validated by this statistical analysis. The normalized hybridization level under 3000 was similar to the macro-array blank value, and the minimal level of significant hybridizations was thus fixed to a normalized value equal to 6000. For each differentially expressed gene, the mRNA number was searched using a BLAST (blastn) program against all available mRNA sequences in NCBI database.

In this investigation, only a subset of genes spotted on the MAIZEWALL macro-array was considered, in order to focus on lignin pathway, cell wall carbohydrate and cell wall protein genes and their transport and regulation factors. This subset thus comprised 105 phenylpropanoid genes, 44 transcription factor genes, 22 auxin related and tissue patterning genes, 33 transport and detoxification process associated genes, 18 cell wall protein genes, and 114 nucleotide sugar and cell wall carbohydrate genes. The exhaustive gene list has been given by Guillaumie et al. [43].

### RT-PCR

Total RNA was isolated as described above. cDNA synthesis was conducted by incubating 1 μl of DNase-treated total RNA (1 μg/μl) with 2 μl of Oligo(dT)15 Primer (Promega, 500 μg/ml) and 15 μl of ultra-pure water for 5 min at 70°C and cooled down on ice for 5 min. 5 μl of Promega's MuMLVRT reverse transcriptase 5× buffer, 1.3 μl of 10 mM dNTP and 200 units of MuMLV-RT reverse transcriptase (Promega) were added to the denatured RNA and incubated at 37°C for 1 h. The reaction was stopped during 5 min at 70°C and then 5 min on ice. PCR was performed as follows: 95°C for 2 min, X cycles (15 cycles for 18S rRNA and 30 cycles for PAL, CCoAOMT3 and COMT) at 95°C for 30s, 55°C–60°C for 30s depending on primer couples, 72°C for 30s and final extension 2 min in a total volume of 25 μl with 12.5 μl of 2× Master Mix PCR (Promega), 1 μl of forward and reverse specific primers of each fragment (10 μM each), 9.5 μl of ultra-pure water and 1 μl cDNA product. Resulting PCR products were analyzed by electrophoresis and stained with Etbr. The PCR primer combinations for each gene were as follows: PAL (2161072.2.1, L77912) forward 5'-GGGGAGGAAATACGTGAAAA-3', reverse 5'-TTAGAAGGAATTGAGTACGC-3'; CCoAOMT3 (2591258.2.1, AY104670) forward 5'-CGTTCACGTCTGCCAGGT-3', reverse 5'-TTCACTCAAGCCCAGTTCG-3'; COMT (2192909.2.3, M73235): forward 5'-GCTTGCTTGGTCCTCGTATC-3', reverse 5'-TACTCGCACATGGCAGAGAC-3'; 18S rRNA: forward 5'-CATGTCAAATTTCACTGCTTCATC-3', reverse 5'-TGACCACCCAGCCATCCTT-3'. All primers were synthesized by MWG-BIOTECH (Ebersberg, Germany).

## Abbreviations

4CL: 4-coumarate:CoA ligase, 5-OH-G: 5-hydroxyguaiacyl, ABC: ATP-binding cassette, AGL: Agamous like, AGP: arabinogalactan protein, ALDH: aldehyde dehydrogenase, ARF: ADP- ribolysation factor, AS225: COMT antisens, *bm*: *brown-midrib*, C3'H: *p*-coumaroyl-shikimate/quinate 3'-hydroxylases, C4H: cinnamate 4-hydroxylase, CAD: cinnamyl alcohol dehydrogenase, CCoAOMT: caffeoyl-CoA O-methyltransferase, CCR: cinnamoyl-CoA reductase, COMT: caffeic acid O-methyltransferase, COV: CONTINUOUS VASCULAR RING, CHI: chalcone flavonone isomerase, DM: dry matter, F5H: ferulate 5-hydroxylase, FA: ferulic acid, G: guaiacyl, GEF: guanine nucleotide exchange factor, GlcA: glucuronic acid, GRP: glycine-rich protein, GST: glutathion S-transferase, GSTs: gene specific tags, H: *p*-hydroxyphenyl, HCT: hydroxycinnamoyl transferase, HD-Zip: Homeodomain-leucine zipper, HRGP: hydroxyproline-rich protein, MP: Monopteros, NDF: neutral detergent fiber, PAL: phenylalanine ammonia-lyase, pCA: *p*-coumaric, PID: PINOID, S: syringyl, PRP: proline-rich protein, SAD: sinapyl alcohol dehydrogenase, SHP: Shatterproof, TAL: tyrosine ammonia-lyase, UDP: uridine diphosphate, XTH: Xyloglucan endotransglycolase/hydrolase.

## Authors' contributions

SG conducted the research, designed the experiments, analyzed the data and drafted the manuscript together with YB. DG and MP coordinated the construction of the MaizeWall macro-array and contributed to the experimental design of this work. OB contributed to acquisition of expression data. YB coordinated the project, and coordinated together with J-PM the Génoplante ZmS3P2 and B5 projects in which this work was managed. All authors read and approved the final manuscript.
